# Long non-coding RNA MCM3AP antisense RNA 1 promotes non-small cell lung cancer progression through targeting microRNA-195-5p

**DOI:** 10.1080/21655979.2021.1950282

**Published:** 2021-08-04

**Authors:** Dijian Shen, Jianqiang Li, Kaiyi Tao, Youhua Jiang

**Affiliations:** a Department of Thoracic Surgery, Cancer Hospital of University of Chinese Academy of Sciences (Zhejiang Cancer Hospital), Hangzhou, China; b Institute of Cancer and Basic Medicine (IBMC), Chinese Academy of Science, Hangzhou, China

**Keywords:** MCM3AP-AS1, miR-195-5p, E2F3, NSCLC

## Abstract

Lung cancer (LC) ranks first among all causes of cancer-related death, with non-small cell lung cancer (NSCLC) taking up 85% of lung cancer cases. Although lncRNA MCM3AP antisense RNA 1 (MCM3AP-AS1) has been reported to be an oncogenic factor in NSCLC, its detailed mechanism in NSCLC is unknown. In this study, quantitative real-time polymerase chain reaction (qRT-PCR) was performed to determine MCM3AP-AS1, microRNA (miR)-195-5p and E2F transcription factor 3 (E2F3) mRNA expressions in NSCLC tissues and cells. Western blot was utilized to determine the expression levels of E2F3, BCL2-associated X protein (Bax), B-cell lymphoma-2 (Bcl-2), E-cadherin and N-cadherin. CCK-8 and Transwell assays were conducted to examine cell proliferation, migration and invasion, respectively. Dual-luciferase reporter assay and RNA immunoprecipitation experiments were used to determine the regulatory relationships between MCM3AP-AS1 and miR-195-5p, and miR-195-5p and E2F3. We demonstrated that MCM3AP-AS1 was overexpressed in NSCLC tissues and cells, and MCM3AP-AS1 overexpression accelerated the proliferation, migration and invasion of NSCLC cells. In addition, MCM3AP-AS1 overexpression markedly up-modulated Bcl-2 expression and repressed Bax expression; MCM3AP-AS1 overexpression also significantly up-regulated N-cadherin expression and suppressed E-cadherin expression in NSCLC cells. What is more, in NSCLC cells, miR-195-5p was a target of MCM3AP-AS1, and the latter worked as a molecular sponge for miR-195-5p to regulate E2F3 expression. Collectively, MCM3AP-AS1, serving as a competitive endogenous RNA (ceRNA) to regulate miR-195-5p/E2F3 axis, promotes NSCLC progression, which is a promising therapeutic target for NSCLC.

## Graphical bastract

**Figure:**
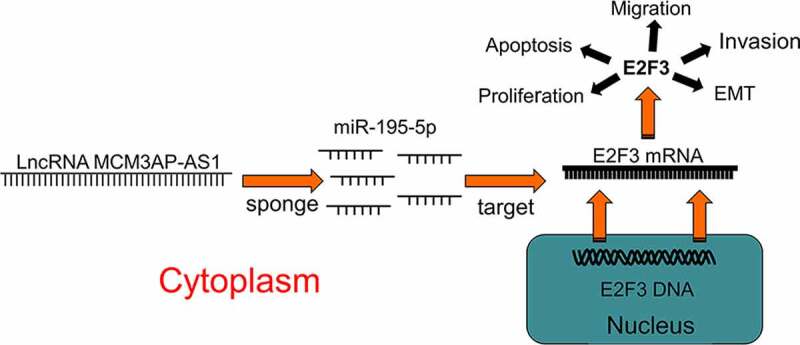

## 1. Introduction

Lung cancer (LC) is the commonest malignancy in the world with the highest mortality [1]. Non-small cell lung cancer (NSCLC), as the main pathological subtype of LC, accounts for more than 80% of cases of LC [2]. The treatment strategies of NSCLC mainly include surgery, radiotherapy, chemotherapy, targeted therapy and immunotherapy [3]. Although a lot of efforts have been made to improve the therapy of NSCLC, the five-year survival rate of the patients is only about 18% [4,5]. Therefore, it is necessary to have a better understanding of the molecular mechanisms underlying NSCLC development to further improve the prognosis of the patients.

In recent years, accumulating studies find that long non-coding RNAs (lncRNAs) are aberrantly expressed in diverse tumors, which is involved in tumorigenesis and disease development [6,7]. For instance, lncRNA TPTEP1, SPRY4-IT1 and ZEB2-AS1 are reported to be aberrantly expressed in NSCLC and modulate biological processes such as proliferation and metastasis of cancer cells [8–10]. Previous researches show that lncRNA MCM3AP antisense RNA 1 (MCM3AP-AS1) is associated with the progression of some tumors, such as prostate carcinoma, gastric carcinoma and cervical carcinoma [11–13]. Reportedly, in NSCLC, MCM3AP-AS1 expression is also up-regulated, and the proliferation, migration and angiogenesis of NSCLC cells are impeded by MCM3AP-AS1 knockdown [14]. However, the detailed mechanism of its effects in NSCLC is vague.

Some previous studies report that microRNA-195-5p (miR-195-5p) is a tumor suppressor in multiple cancers including NSCLC [15–17]. Interestingly, our bioinformatics data suggested that MCM3AP-AS1 contained the binding sequence for miR-195-5p. We supposed that MCM3AP-AS1 could probably contribute to NSCLC progression via interacting with miR-195-5p. In the current research, we validated that MCM3AP-AS1 expression was up-regulated in NSCLC tissues and MCM3AP-AS1 enhanced the proliferation and metastasis of NSCLC cells; mechanistically, it was unmasked that MCM3AP-AS1 exerted tumor-promoting effects by modulating the miR-195-5p/E2F transcription factor 3 (E2F3) axis.

## 2. Materials and methods

### 2.1. Sample collection

From June 2017 to August 2018, sixty-three pairs of matched NSCLC tissues and tissues of resection margin were taken from the tissue bank of the Cancer Hospital of the University of Chinese Academy of Sciences. All patients were histologically confirmed as NSCLC by pathologists at the Cancer Hospital of the University of Chinese Academy of Sciences and were not suffering from any other malignancies, and no neoadjuvant therapy was given prior to surgery. Among the patients, there are 38 males and 25 females, aged from 41–74 years old (average: 52.4). Patients receiving neoadjuvant therapy, patients with mixed NSCLC/small cell histology or CNS metastases were excluded. The surgically resected NSCLC tissues and paracancerous tissues (at least 5 cm away from the tumor margin) were collected and stored in liquid nitrogen immediately after resection during surgery. All specimens were confirmed as NSCLC or normal lung tissue by postoperative pathological examination. All patients who provided tissues signed informed consent before the surgery, and all of them agreed to the use of their samples in scientific research. This research was endorsed by the Research Ethics Committee of Cancer Hospital of the University of Chinese Academy of Sciences (Approval Number: 2017–05).

### 2.2. Cell culture

NSCLC cells (A549, H358, H1299, H460 and H226) and human lung epithelial cells BEAS-2B were available from China Center for Type Culture Collection (CCTCC, Wuhan, China). The cells were cultured in RPMI-1640 medium (Biosharp, Shanghai, China) containing 10% fetal bovine serum (FBS, Biosharp, Shanghai, China) at 37°C in 5% CO_2_.

### 2.3. Cell transfection

MiR-195-5p mimics (5ʹ-UAGCAGCACAGAAAUAUUGGC-3ʹ) and its control miR NC (5ʹ-UCACAACCUCCUAGAAAGAGUAGA-3ʹ), miR-195-5p inhibitors (5ʹ-GCCAAUAUUUCUGUGCUGCUA-3ʹ) and its control inh NC (5ʹ-UUGUACUACACAAAAGUACUG-3ʹ), two different shRNAs targeting MCM3AP-AS1 (sh-MCM3AP-AS1#1: 5ʹ-GCTCACAATGATGGCACTA-3ʹ and sh-MCM3AP-AS1#2:

5ʹ-GGACAGAGGGAACATGGAT-3ʹ), its control sh-NC (5ʹ-GCTTAACGTAGACGACCTA-3ʹ), MCM3AP-AS1 overexpression plasmid and their negative controls (GenePharma, Shanghai, China) were designed and synthesized. In transfection, the final concentration of oligonucleotides was 50 nM. Transfection was conducted using Lipofectamine 2000 (Invitrogen, Carlsbad, CA, USA) under the guidance of the protocol provided by the manufacturer. After 24 h following transfection, transfection efficiency was determined using quantitative real-time polymerase chain reaction (qRT-PCR) or used for subsequent experiments.

### 2.4. Bioinformatics analysis

In this study, GEPIA database (http://gepia.cancer-pku.cn/) was searched to analyze MCM3AP-AS1 expression in NSCLC samples [18]; Kaplan Meier-plotter database (https://kmplot.com/) was employed to analyze the prognostic prediction value of MCM3AP-AS1 [19]; StarBase database (http://starbase.sysu.edu.cn/) was used to validate the correlations among MCM3P-AS1, miR-195-5p and E2F3 [20]. LncBase Predicted v.2 (http://carolina.imis.athena-innovation.gr/diana_tools/web/) [21] and TargetScan databases (http://www.targetscan.org/vert_72/) [22] were applied to look for the binding sites between miR-195-5p and MCM3AP-AS1, E2F3 3ʹUTR.

### 2.5. qRT-PCR

Total RNA from tissues and cells was isolated using TRIzol reagent (Invitrogen, Carlsbad, CA, USA). Total RNA was reversely transcribed into cDNA using the reverse transcription kit (Applied Biosystems, Foster City, CA, USA). cDNA was used as a template, and qRT-PCR was conducted using SYBR® Premix-Ex-Taq™ (Takara, Tokyo, Japan) on ABI7300 PCR system (Thermo Fisher Scientific, Waltham, MA, USA). Additionally, the relative expression of miR-195-5p was normalized to U6, and the relative expressions of MCM3AP-AS1 and E2F3 mRNA were normalized to GAPDH, with 2^−ΔΔCt^ method [23]. Primer sequences were shown in Table 1.

**Table 1.** Sequences used for qRT-PCR

MCM3AP-AS1	F: GCTGCTAATGGCAACACTGA R: AGGTGCTGTCTGGTGGAGAT
miR-195-5p	F:ACACTCCAGCTGGGTAGCAGCACAGAAAT
	R: TGGTGTCGTGGAGTCG
E2F3	F: AGAAAGCGGTCATCAGTACCT
	R: TGGACTTCGTAGTGCAGCTCT
U6	F: GCTTCG GCAGCACATATACTAAAAT
	R: CGCTTCACGAA TTTGCGTGTCAT
GAPDH	F: GTCGATGGCTAGTCGTAGCATCGAT
	R: TGCTAGCTGGCATGCCCGATCGATC

### 2.6. Cell viability

A549 and H226 cells were inoculated into 96-well plates at 1.0 × 10^3^/well before 10 μL of cell counting kit (CCK-8) reagent (MedChem Express, Monmouth Junction, New Jersey, USA) was added into the wells at the 1st, 2nd, and 3rd d, respectively, and OD 450 values were measured on a microplate reader after 2 h of incubation.

### 2.7. EdU assay

A549 and H226 cells were inoculated into 24-well plates, respectively, and cultured for 24 h. After that, the cells were incubated with 200 μL of 50 μmol/L EdU medium (RiboBio Co., LTD, Guangzhou, China) for 2 h, rinsed with PBS and then fixed with paraformaldehyde for 10 min. Following that, the cells were incubated for 5 min with 200 μL of 2 mg/ml of glycine and rinsed with PBS for 5 min. One hundred microliters of PBS with 0.5% Triton X-100 was added to each well, and the cells were placed on a shaker for 10 min and then washed with PBS for 5 min. Subsequently, the cells were stained with Apollo® solution in the dark for 30 min, and nuclear DNA was counterstained with DAPI. Then, the cells were observed, and the images were collected using a fluorescent microscope in the dark.

### 2.8. Transwell assay

Migration assays were performed using Transwell chambers (8 µM pore size; Corning, Beijing, China). After dispersing A549 and H226 cells with 0.25% trypsin, the cells were centrifuged and resuspended with serum-free medium. 5 × 10^4^ cells were inoculated into each chamber before the complete medium was added in the 24-well plate. After 24 h incubation at 37°C, the unmigrated cells were removed. The cells on the Transwell membranes were fixed with 4% paraformaldehyde for 10 min and stained with 0.5% crystal violet solution. After being rinsed with tap water, the migrated cells were counted under an inverted microscope. Invasion experiments were performed with Transwell chambers coated with Matrigel, and the remaining procedures were the same as the migration assay.

### 2.9. Western blot

Cellular protein was extracted using RIPA lysis buffer (Beyotime Biotechnology, Shanghai, China), and protein concentration was determined using a BCA kit (Beyotime Biotechnology, Shanghai, China). After SDS-PAGE, the protein samples were transferred to PVDF membranes and blocked with 5% skimmed milk for 1 h at room temperature. The corresponding primary antibodies were then added to interact with the proteins at 4°C overnight, and then the membrane was rinsed with tris buffered saline with Tween 20 (TBST). Next, the secondary antibodies were added to incubate the membrane for 2 h at room temperature before the membrane was rinsed with TBST again, and then ECL chemiluminescent kit (Millipore, Billerica, MA, USA) was added onto the membrane to develop the bands. The antibodies used in this study, including anti-E2F3 (ab50917, 1:500), anti-Bax (ab32503, 1:500), anti-Bcl-2 (ab185002, 1:500), anti-E-cadherin (ab11512, 1:500), anti-N-cadherin (ab18203, 1:500) and anti-β-actin (ab179467, 1:2000), were all bought from Abcam (Shanghai, China).

### 2.10. Dual-luciferase reporter gene experiment

Wild-type MCM3AP-AS1 and mutant MCM3AP-AS1 sequence, or wild-type E2F3 3′-UTR and mutant E2F3 3′-UTR sequence were inserted into a pGL3 vector (Promega, Madison, WI, USA) to construct MCM3AP-AS1-WT1, MCM3AP-AS1-WT2, MCM3AP-AS1-WT3, MCM3AP-AS1-MUT1, MCM3AP-AS1-MUT2, MCM3AP-AS1-MUT3, E2F3-WT and E2F3-MUT luciferase reporter vectors. The luciferase reporter experiment was conducted using the dual-luciferase reporter assay kit (Promega, Madison, WI, USA). MCM3AP-AS1-WT, MCM3AP-AS1-MUT, E2F3-WT and E2F3-MUT were co-transfected into HEK-293 T cells with miR-195-5p mimic (5ʹ-UAGCAGCACAGAAAUAUUGGC-3ʹ), miR-NC (5ʹ-UCACAACCUCCUAGAAAGAGUAGA-3ʹ), miR-195-5p inhibitors (5ʹ-GCCAAUAUUUCUGUGCUGCUA-3ʹ) and inh-NC (5ʹ-UUGUACUACACAAAAGUACUG-3ʹ) using Lipofectamine 2000 (Invitrogen, Carlsbad, CA, USA), respectively, and the luciferase activity of the reporter of each group was measured 24 h later. The relative luciferase activity was normalized with renilla luciferase activity.

### 2.11. RIP experiment

A549 and H226 cells were transfected with pcDNA3.1-MCM3AP-AS1 vector or pcDNA3.1 vector. Forty-eight hours later, the cells were subjected to RIP assays using anti-Ago2 antibody and the Magna RIP™ RNA-Binding Protein Immunoprecipitation Kit (Millipore, Billerica, MA, USA) according to the manufacturer’s instruction. qRT-PCR was conducted to detect MCM3AP-AS1 and miR-195-5p expressions in the immunoprecipitate.

### 2.12. Lung metastasis model in vivo

The animal experiments were approved by the Animal Research Ethics Committee of Cancer Hospital of the University of Chinese Academy of Sciences (Approval Number: 2020–06). Ten male BALB/c nude mice (6–8 weeks old, average weight: 24.5 g ± 2.20) were purchased from Zhejiang Province Experimental Animal Center (Hangzhou, China). Mice were maintained under standard housing conditions (23°C, 40% humidity, 12 h light cycles, and free access to food and water). The mice were randomly divided into two groups (NC group and MCM3AP-AS1 overexpression group, n = 5 in per group). A549 cells (2 × 10^7^ cells/per mouse) were injected into each mouse via tail vein. Three weeks later, the mice were euthanized with 100% oxygen/5% isoflurane, and the bilateral thoracotomy was used to confirm the death. Lung tissues were removed after lavage, fixed in 10% neutral formalin for 36 hours, and embedded in paraffin. Then, hematoxylin/eosin staining was performed for pathological examination of lung metastatic nodules of the mice. The number of pulmonary metastatic nodules in each section was counted in 5 randomly selected visual fields under the microscope (Olympus, Tokyo, China).

### 2.13. Statistical analysis

All the experiments were conducted in triplicate. The data were processed by GraphPad Prism 8.0 (GraphPad Software, Inc., La Jolla, CA, USA), plotted and represented as mean ± standard deviation. To make the comparison between two groups, One-Sample Kolmogorov–Smirnov test was used to examine whether the data are normally distributed. For the data which were normally distributed, independent sample t test was used. For the data which were skewed distributed, paired sample Wilcoxon signed rank test was used. One-way ANOVA test was performed to make the comparison among three or more groups. If there was a significant difference, Newman–Keuls analysis was performed to make the comparison between two groups. Pearson correlation analysis was used to determine the correlation. *P* < 0.05 signified statistical significance.

## 3. Results

In this study, we performed a series of experiments to investigate the biological function of MCM3AP-AS1 in NSCLC progression, and explore the regulatory mechanism of MCM3AP-AS1/miR-195-5p/E2F3 axis, which were aimed to help clarify the mechanism of NSCLC progression.

### 3.1. MCM3AP-AS1 and E2F3 expressions were up-regulated, and miR-195-5p expression was down-regulated in NSCLC tissue specimens

**Figure 1.**
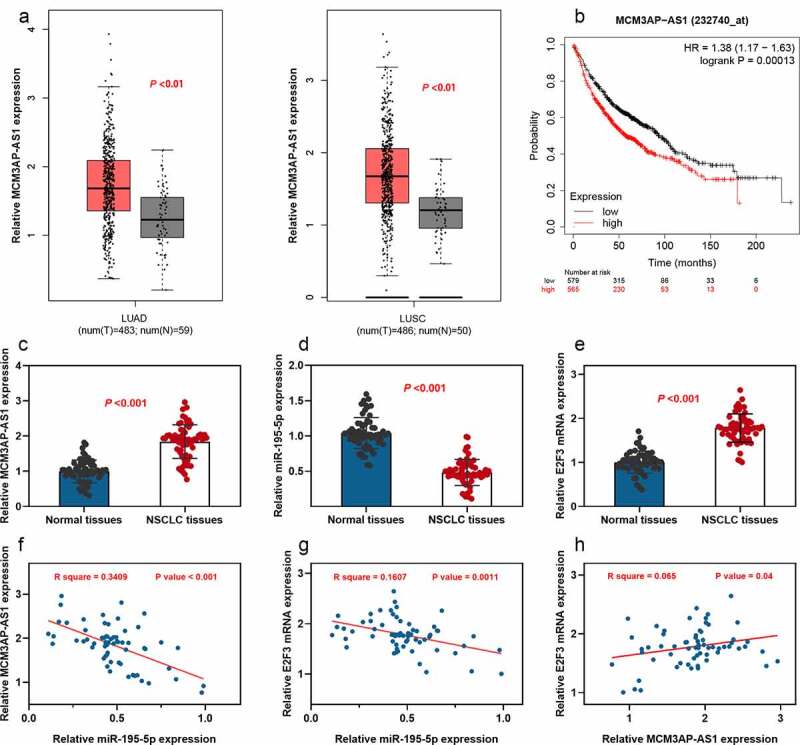
Expression of MCM3AP-AS1, miR-195-5p and E2F3 in NSCLC

First of all, gene expression analysis and survival analysis were conducted using the GEPIA database. As shown, MCM3AP-AS1 expression was up-regulated in both lung adenocarcinoma tissues (LUAD) and lung squamous carcinoma (LUSC) tissues; additionally, MCM3AP-AS1 overexpression was significantly linked to a shorter overall survival time of NSCLC patients (Figure 1a–b). Then, the expression patterns of MCM3AP-AS1, miR-195-5p and E2F3 in paired specimens collected from 63 patients with NSCLC were examined by qRT-PCR, respectively. We found significant up-regulation of both MCM3AP-AS1 and E2F3 expressions and downregulation of miR-195-5p expression in NSCLC tissue specimens, in comparison with these in paired adjacent normal tissues (Figure 1c–e). Pearson’s correlation analysis further indicated that MCM3AP-AS1 and E2F3 were negatively correlated with miR-195-5p; conversely, MCM3AP-AS1 expression was positively correlated with E2F3 expression in NSCLC samples (Figure 1f–h). StarBase database also suggested that MCM3AP-AS1 expression and E2F3 expression were negatively correlated with miR-195-5p expression in NSCLC samples; conversely, MCM3AP-AS1 expression was positively correlated with E2F3 expression (Supplementary Figure 1a-c). Moreover, statistically, high MCM3AP-AS1 expression was closely associated with the larger tumor size, low differentiation and higher TNM stage of the NSCLC patients (Table 2). These results suggested that MCM3AP-AS1 was an oncogenic factor for NSCLC and might have regulatory functions on miR-195-5p and E2F3.

**Table 2.** Correlation between clinicopathological features and expression of MCM3AP-AS1 in NSCLC

Pathological parameters	Numbers (n = 63)	MCM3AP-AS1 expression High (n = 32) Low (n = 31)		χ2	*p-*Value
Gender				0.7654	0.3817
Male	38	21	17		
Female	25	11	14		
Age (years)				0.0186	0.8915
<50	29	15	14		
≥50	34	17	17		
Smoking				1.3548	0.2444
Nonsmoker	36	16	20		
Smoker	27	16	11		
Tumor size (cm)				4.5853	0.0322*
<5	28	10	18		
>5	35	22	13		
TNM stage				5.8572	0.0156*
I+II	25	8	17		
III+IV	38	24	14		
Degree of differentiation				5.7634	0.0164*
Low,medium	36	23	13		
High	27	9	18		

*P < 0.05.

(a) Analysis of MCM3AP-AS1 expression in NSCLC tissues (LUAD: n = 483; LUSC: n = 486) and non-cancerous tissues (N = 50) was conducted using the online database GEPIA (Match TCGA normal data). (b) Kaplan–Meier plotter database was used to conduct Kaplan–Meier analysis of the overall survival time of patients with NSCLC with high (n = 565) and low (n = 579) MCM3AP-AS1 expression levels (Affy ID: 232740_at; the samples were divided into high and low expression groups according to the median value of MCM3AP-AS1 expression). (c) qRT-PCR was performed to detect MCM3AP-AS1 expressions in 63 cases of NSCLC tissues and adjacent tissues. (d) qRT-PCR was performed to detect miR-195-5p expressions in 63 cases of NSCLC tissues and adjacent tissues. (e) qRT-PCR was performed to detect E2F3 mRNA expressions in 63 cases of NSCLC tissues and adjacent tissues. (f–h) Pearson’s correlation analysis was performed to assess the correlations among MCM3AP-AS1, miR-195-5p and E2F3 mRNA in NSCLC tissues.

### 3.2. MCM3AP-AS1 repressed miR-195-5p expression in NSCLC cells

**Figure 2.**
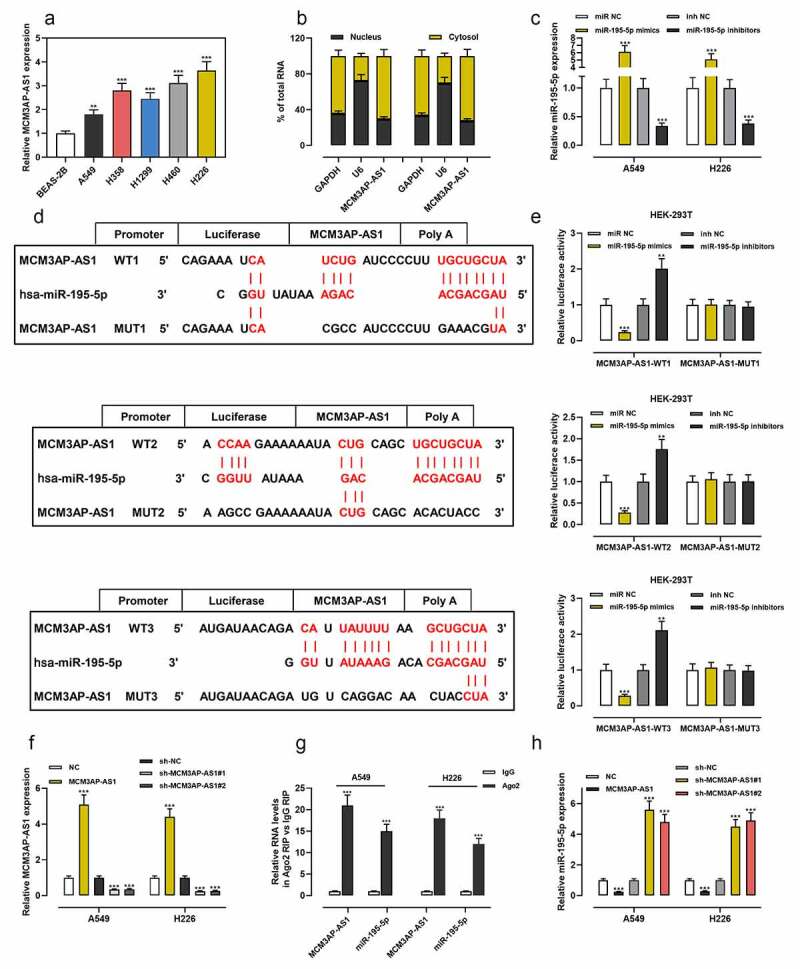
MCM3AP-AS1 was specifically regulated by miR-195-5p

Then, we found that MCM3AP-AS1 was overexpressed in NSCLC cell lines (Figure 2a). Importantly, lncRNAs usually function as competitive endogenous RNA (ceRNA) by binding to miRNAs and can inhibit the expressions of miRNAs. We demonstrated that MCM3AP-AS1 was enriched in the cytoplasm but not in the nucleus of NSCLC cells (Figure 2b). MiR-195-5p expression in cells was much higher after the transfection of miR-195-5p mimics than the transfection with miR-NC; miR-195-5p expression was much lower after the transfection with miR-195-5p inhibitors than the transfection with inhibitors-NC (Figure 2c). Bioinformatics analysis predicted that there were three potential binding sites for miR-195-5p in the sequence of MCM3AP-AS1 (Figure 2d). Dual-luciferase reporter experiment revealed that miR-195-5p mimics weakened the luciferase activity of the MCM3AP-AS1-WT reporter vectors, while miR-195-5p inhibitors increased it, but miR-195-5p mimics or inhibitors had no significant effects on the MCM3AP-AS1-MUT reporter vectors (Figure 2e). Next, MCM3AP-AS1 overexpression plasmid, sh-MCM3AP-AS1#1 and sh-MCM3AP-AS1#2 were transfected into NSCLC cells, and qRT-PCR verified the success of transfection (figure 2f). The RIP experiment demonstrated that MCM3AP-AS1 and miR-195-5p were remarkably enriched in Ago2-immunoprecipitates compared with the control group (Figure 2g). Moreover, MCM3AP-AS1 overexpression decreased miR-195-5p expression, while MCM3AP-AS1 knockdown worked oppositely in NSCLC cells (Figure 2h).

(a) MCM3AP-AS1 expressions in BEAS-2B cells and NSCLC cells were measured by qRT-PCR. (b) Subcellular localization of MCM3AP-AS1 in A549 and H226 was assessed by qRT-PCR after nuclear–cytoplasm fractionation. (c) MiR-195-5p mimics or miR-195-5p inhibitors were transfected into A549 and H226 cells, respectively, and the transfection efficiency was examined by qRT-PCR. (d) The schematic map of the MCM3AP-AS1 WT and MCM3AP-AS1 MUT binding sites for miR-195-5p, which was predicted by LncBase Predicted v.2 (Score: 0.947). (e) MCM3AP-AS1-WT (WT1, WT2 and WT3) or MCM3AP-AS1-MUT (MUT1, MUT2 and MUT3) was co-transfected into HEK-293 T cells with miR-195-5p mimics or miR-195-5p inhibitors, and the relative luciferase activity was measured. (f) Transfection efficiency of MCM3AP-AS1 overexpression plasmids, sh-MCM3AP-AS1#1 or sh-MCM3AP-AS1#2 was detected by qRT-PCR. (g) The interaction between MCM3AP-AS1 and miR-195-5p in A549 and H226 cells was analyzed by RIP experiment. (h) Effect of MCM3AP-AS1 knockdown and overexpression on miR-195-5p expression in A549 and H226 cells was detected by qRT-PCR. All of the experiments were performed in triplicate. ** *P* < 0.01 and *** *P* < 0.001.

### 3.3. The effect of MCM3AP-AS1/miR-195-5p axis on the proliferation of NSCLC cells

**Figure 3.**
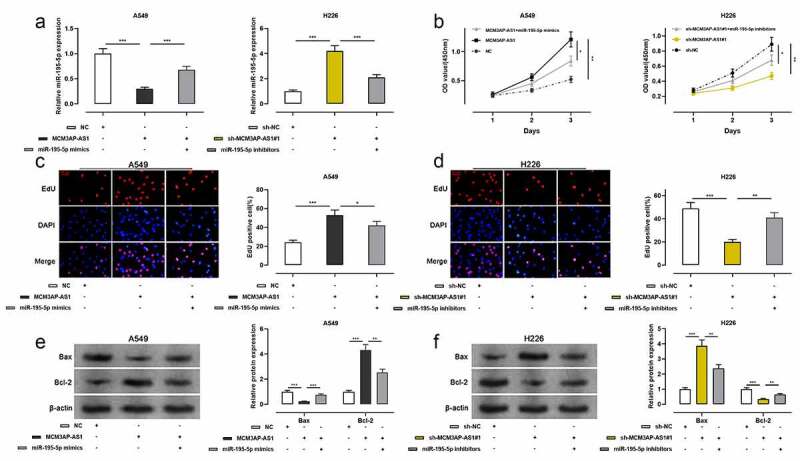
Effect of MCM3AP-AS1 and miR-195-5p on A549 and H226 cells proliferation and apoptosis

To clarify the function of MCM3AP-AS1 in NSCLC progression, A549 cells were co-transfected with MCM3AP-AS1 overexpression plasmid and miR-195-5p mimics; H226 cells were co-transfected with sh-MCM3AP-AS1#1 and miR-195-5p inhibitors, and qRT-PCR showed that the transfection was successful (Figure 3a). The proliferation of these two cell lines was assessed by CCK-8 and EdU assays, and the data presented that the proliferation of NSCLC cells was markedly increased after the transfection with MCM3AP-AS1 overexpression plasmid, while miR-195-5p mimics abolished this effect; the proliferation of NSCLC cells was remarkably decreased after the transfection with sh-MCM3AP-AS1#1, while co-transfection of miR-195-5p inhibitors reversed this effect (Figure 3b–d). Western blot assay suggested that after MCM3AP-AS1 overexpression, Bcl-2 protein level was increased and BAX protein level was decreased, while miR-195-5p mimics counteracted such effect; after knockdown MCM3AP-AS1, Bcl-2 protein level was declined and Bax protein level was elevated, while miR-195-5p inhibitors reversed this effect (Figure 3e–f). These results suggested that MCM3AP-AS1 promoted the growth and repressed the apoptosis of NSCLC cells, which was mediated by its inhibitory function on miR-195-5p.

(a) MCM3AP-AS1 overexpressing plasmid and miR-195-5p mimics, sh-MCM3AP-AS1#1 and miR-195-5p inhibitors were co-transfected into A549 and H226 cells, respectively. qRT-PCR was used to detect transfection efficiency. (b) After the transfection, CCK-8 method was applied to detect the cell viability of A549 and H226 cells. (c–d) After the transfection, EdU assay was utilized to detect the proliferation of A549 and H226 cells. (e–f) After the transfection, Western blot was employed to detect the Bax and Bcl-2 expressions. All of the experiments were performed in triplicate. *P < 0.05, **P < 0.01 and *** *P* < 0.001.

### 3.4. The effect of MCM3AP-AS1/miR-195-5p axis on the metastasis of NSCLC cells

**Figure 4.**
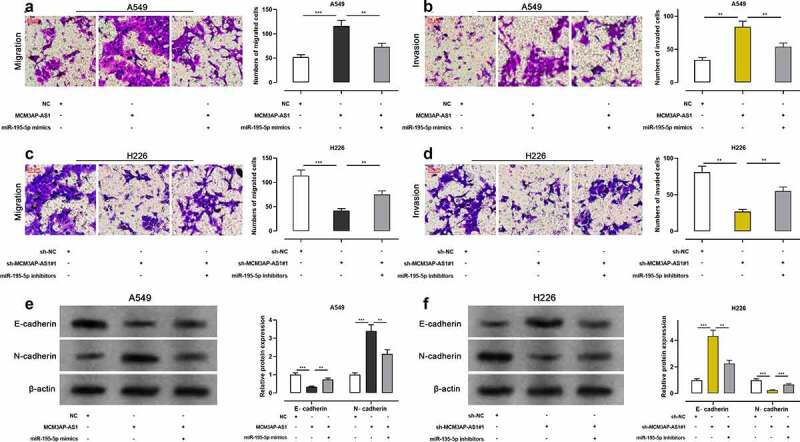
Effect of MCM3AP-AS1 on NSCLC cell migration and invasion

The migration and invasion of these two cell lines were assessed using Transwell assay, and the results showed that the number of migrated and invaded cells was significantly raised after NSCLC cells were transfected with MCM3AP-AS1 overexpression plasmid, while miR-195-5p mimics abrogated such effect (Figure 4a–b); the number of migrated and invaded cells was markedly reduced after the transfection with sh-MCM3AP-AS1#1 while miR-195-5p inhibitors reversed such effect (Figure 4c–d). After MCM3AP-AS1 overexpression, E-cadherin protein level was decreased and N-cadherin protein level was increased, while miR-195-5p mimics reversed this effect; after knockdown MCM3AP-AS1, E-cadherin protein level was elevated and N-cadherin protein level was declined, while miR-195-5p inhibitors reversed such effect (Figure 4e–f). Lung metastasis experiments in nude mice indicated that overexpression of MCM3AP-AS1 promoted lung metastasis *in vivo* (Supplementary Figure 1d). These results manifested that MCM3AP-AS1 facilitated the metastasis of NSCLC, which was mediated by its inhibitory function on miR-195-5p.

(a–d) MCM3AP-AS1 overexpressing plasmid and miR-195-5p mimics, sh-MCM3AP-AS1#1 and miR-195-5p inhibitors were co-transfected into A549 and H226 cells, respectively. Transwell experiment was performed to detect the migration and invasion of A549 and H226 cells. (e–f) MCM3AP-AS1 overexpressing plasmid and miR-195-5p mimics, sh-MCM3AP-AS1#1 and miR-195-5p inhibitors were co-transfected into A549 and H226 cells, respectively. Western blot was conducted to detect the expression of E-cadherin and N-cadherin in A549 and H226 cells. All of the experiments were performed in triplicate. ** *P* < 0.01, *** *P* < 0.001.

### 3.5. MCM3AP-AS1 regulated E2F3 expression by decoying miR-195-5p

**Figure 5.**
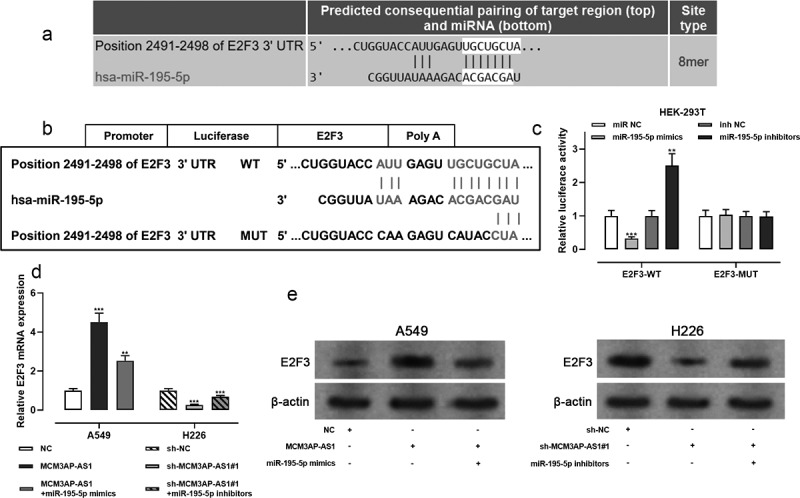
MCM3AP-AS1 up-regulated E2F3 expression by adsorbing miR-195-5p

TargetScan database indicated that miR-195-5p could probably target the 3ʹ-UTR of E2F3 (Figure 5a), and this prediction was validated by dual-luciferase reporter assay. The data illustrated that miR-195-5p mimics reduced the luciferase activity of the E2F3-WT reporter vector, while miR-195-5p inhibitors functioned oppositely; miR-195-5p mimics and inhibitors had no significant effects on E2F3-MUT reporter vector (Figure 5b–c). qRT-PCR and Western blot showed that overexpression of MCM3AP-AS1 promoted E2F3 expression, while miR-195-5p mimics counteracted the function of MCM3AP-AS1 in NSCLC cells; conversely, MCM3AP-AS1 knockdown inhibited E2F3 expression, while miR-195-5p inhibitors reversed such effect (Figure 5d–e). These results suggested that MCM3AP-AS1, as a ceRNA for miR-195-5p, could induce the expression of E2F3 by repressing miR-195-5p expression.

(a–b) The schematic map of the E2F3 3ʹUTR WT and E2F3 3ʹUTR MUT binding site for miR-195-5p, which was predicted by TargetScan database (Context score percentile: 95). (c) E2F3 3ʹUTR-WT or E2F3 3ʹUTR-MUT was co-transfected into HEK-293 T cells with miR-195-5p mimics or miR-195-5p inhibitors, and the relative luciferase activity was measured. (d–e) MCM3AP-AS1 overexpressing plasmid and miR-195-5p mimics, sh-MCM3AP-AS1#1 and miR-195-5p inhibitors were co-transfected into A549 and H226 cells, respectively. qRT-PCR and Western blot were used to detect the expression of E2F3 in NSCLC cells. All of the experiments were performed in triplicate. ** *P* < 0.01, *** *P* < 0.001.

## 4. Discussion

LncRNA can regulate the expressions of downstream genes at the different levels, including chromatin modification, transcription or post-transcription, thus participating in various biological processes, including cell proliferation, migration and apoptosis [24,25]. The role of lncRNA in cancer biology is reported in a lot of previous research. For example, in NSCLC, lncRNA KCNQ1OT1 and AWPPH expressions are significantly up-modulated in tumor tissues, and their high expressions were correlated to adverse prognosis; KCNQ1OT1 and AWPPH can enhance the proliferation, migration and invasion of NSCLC cells, respectively [26,27]. Reportedly, MCM3AP, which serves as an essential modulator in DNA replication by acetylating micro-chromosome maintenance protein 3 (MCM3), impedes cell cycle progression and modulates gene expression in tumors [28]. MCM3AP-AS1 is the lncRNA antisense of MCM3AP gene. MCM3AP-AS1 is overexpressed in hepatocellular carcinoma tissues and MCM3AP-AS1 knockdown inhibits the malignant phenotypes of hepatocellular carcinoma cells; mechanistically, MCM3AP-AS1 promotes epidermal growth factor receptor (EGFR) expression by the adsorption of miR-455, which in turn enhances the metastasis of hepatocellular carcinoma [29]. Another study reveals that MCM3AP-AS1 regulates the progression of hepatocellular carcinoma via miR-194-5p/FOXA1 axis [30]. In NSCLC, MCM3AP-AS1 knockdown impedes cell proliferation and migration, and YY1 transcription factor (YY1) mediates the transcription of MCM3AP-AS1 in NSCLC [14]. In this study, it was verified that MCM3AP-AS1 expression was up-modulated in NSCLC, and its overexpression was associated with unfavorable prognosis of patients and MCM3AP-AS1 facilitated the proliferation and metastasis of NSCLC cells. Our data prove that MCM3AP-AS1 is a promising biomarker and therapy target for NSCLC, which is consistent with the previous reports [14].

MiRNAs also participate in regulating diverse biological process. Accumulating studies elucidate that multiple miRNAs are aberrantly expressed in diverse tumors, including NSCLC [31–35]. Reportedly, miR-195-5p expression is down-modulated in esophageal carcinoma and miR-195-5p represses the proliferation and metastasis of cancer cells via targeting Fos-related antigen 1 (FOSL1) [15]. In NSCLC, miR-195-5p expression is also down-regulated, and *in vitro* experiments unmask that miR-195-5p impedes the proliferation of NSCLC cells [16]. Additionally, down-regulation of miR-195-5p expression in NSCLC is closely associated with increased TNM staging, increased tumor size and lymph node metastasis; functionally, miR-195-5p suppresses cell proliferation and induces cell cycle arrest and apoptosis [17]. LncRNA can adsorb miRNA as ceRNA and thus participate in tumorigenesis and development [36,37]. For instance, in glioma and HCC, miR-195-5p is sponged by LINC00473 and lncRNA SNHG1, respectively [38,39]. The current study confirmed that miR-195-5p was a downstream target of MCM3AP-AS1 and MCM3AP-AS1 negatively regulated its expression; functional experiments suggested that MCM3AP-AS1/miR-195-5p axis regulated the proliferation and metastasis of NSCLC cells. To the best of our knowledge, our study is the first to validate the interaction between MCM3AP-AS1 and miR-195-5p in cancer biology.

E2F3, which is an important cell cycle regulator and figures prominently in regulating cell proliferation, apoptosis and differentiation, belongs to the E2F transcriptional regulatory family and is closely related to tumorigenesis [40–42]. Reportedly, E2F3 expression is up-regulated in tumors, such as osteosarcoma and breast cancer, and enhances the proliferation and colony formation of tumor cells [42,43]. In NSCLC, E2F3 improves the malignancy of cancer cells by increasing the expressions of cyclinD1, cyclinD2, and CDK4 while inhibiting p21 and p57 expressions [44,45]. The present work verified the binding sites between miR-195-5p and E2F3 3ʹUTR and proved that MCM3AP-AS1 could up-regulate E2F3 expression by adsorbing miR-195-5p, which explained the mechanism of E2F3 dysfunction in NSCLC. Importantly, our data also suggest that MCM3AP-AS1, miR-195-5p and E2F3 form a novel ceRNA network to take part in regulating NSCLC progression.

## 5. Conclusion

MCM3AP-AS1 plays a promotive role in NSCLC progression via modulating the miR-195-5p/E2F3 axis, indicating that MCM3AP-AS1 is a new target for gene therapy in NSCLC. However, this work has several limitations. First, the value of MCM3AP-AS1 as a biomarker to predict the prognosis of NSCLC patients’ needs to be further verified by a larger cohort of patients from different medical centers. Second, whether MCM3AP-AS1 can promote other malignant phenotypes of NSCLC cells, such as drug resistance and radioresistance, needs further investigation. Furthermore, there are other potential downstream miRNAs of MCM3AP-AS1 remained to be screened and validated in the future.

## Supplementary Material

Supplemental Material

## Data Availability

The data used to support the findings of this study are available from the corresponding author upon request.

## Research highlights

1. MCM3AP-AS1 is up-regulated in NSCLC tissues;
2. MCM3AP-AS1 promotes the malignant biological behaviors of NSCLC cells such as proliferation, migration and invasion.
3. The functions of MCM3AP-AS1 are partly dependent on miR-195-5p/E2F3 axis in NSCLC.
