# Supplementary material for: Long non-coding RNA MCM3AP antisense RNA 1 promotes non-small cell lung cancer progression through targeting microRNA-195-5p
Source: Bioengineered. 2021 Aug 4;12(1):3525–38. doi: 10.1080/21655979.2021.1950282 (PMC8806479; doi:10.1080/21655979.2021.1950282)
Supplement: Supplemental Material [file KBIE_A_1950282_SM0077.zip › Supplementary Materialsclean.docx]

**
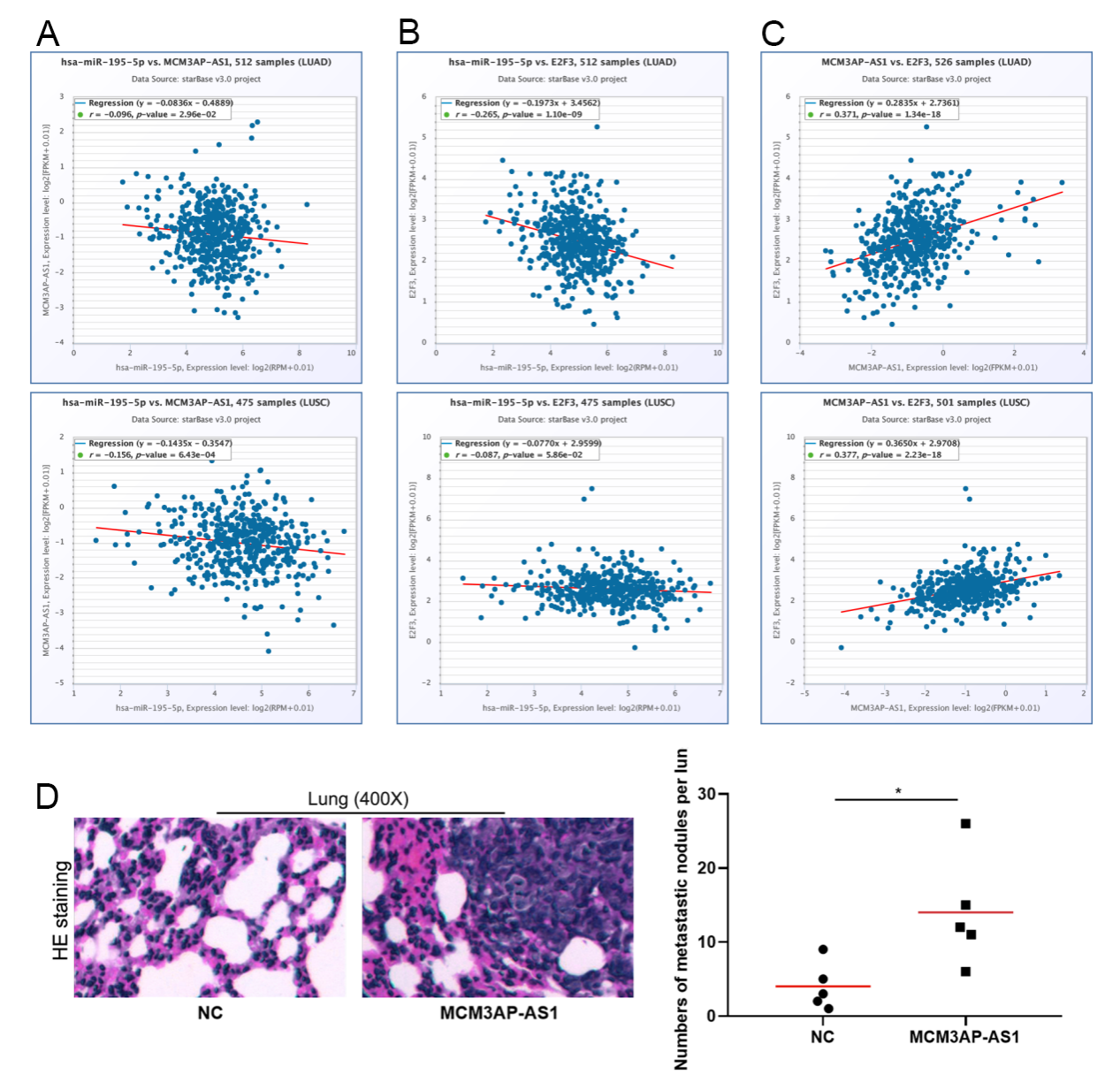
**

**Supplementary Fig. 1 The effect of MCM3AP-AS1 on tumor metastasis in vivo.**

A. Correlation between MCM3AP-AS1 and miR-195-5p expression in NSCLC samples, which was analyzed by StarBase database (Data Source: StarBase v3.0 project, LUAD and LUSC).

B. Correlation between E2F3 mRNA and miR-195-5p expression in NSCLC samples, which was analyzed by StarBase database (Data Source: StarBase v3.0 project, LUAD and LUSC).

C. Correlation between MCM3AP-AS1 and E2F3 mRNA expression in NSCLC samples, which was analyzed by StarBase database (Data Source: StarBase v3.0 project, LUAD and LUSC).

D. Lung metastasis of NSCLC cells *in vivo* was evaluated after the nude mice were injected with A549 cells (NC group or MCM3AP-AS1 overexpression group).
